# Thermal indices for human biometeorology based on Python

**DOI:** 10.1038/s41598-023-47388-y

**Published:** 2023-11-27

**Authors:** Yung-Chang Chen

**Affiliations:** Research Center for Environmental Changes, Academia Sinica, RCEC Building, 128 Academia Road, Section 2, Nankang, Taipei, 115201 Taiwan

**Keywords:** Atmospheric science, Environmental impact, Public health

## Abstract

Thermal indices, such as Predicted Mean Vote, Outdoor Standard Effective Temperature, Physiologically Equivalent Temperature, and Universal Thermal Climate Index, are essential for the evaluation of thermal perception, the design of climate sensitive buildings or urban area, and tourism. These thermal indices are built on complicated numeric models. RayMan was developed to calculate thermal indices based on Delphi program language on the Windows 7 operating system. RayMan is not currently under active maintenance or development. Thus, this report describes the development of an innovative Python library named biometeo that includes an innovative thermal index (modified Physiologically Equivalent Temperature) as a next generation program to calculate thermal indices and human biometeorological variables.

## Introduction

Human biometeorology is experiencing a resurgence in research interest because of concerns about the impact of weather and climate on living organisms and the effects of human activities on the atmospheric environment^1^. The methods used in modern human biometeorology are increasingly diffused among practitioners in related fields, such as urban or regional planners, air conditioning engineers, public and occupant health, and tourism^2–9^. Thermal indices are used to conduct objective investigations into thermal perception as a part of modern human biometeorology^7,10,11^. Thermal indices are also used for the effective and comprehensive evaluation of climate sensitive designs towards the development of a zero-net power grid, sustainable urban development^12–17^, and architectural design^18–20^ for thermal comfort^8,21,22^. Parts of thermal indices are pointed out as standard reference for evaluation of thermal environments for comfort^23,24^. Thermal indices can be categorized as empirical or rational^10,25^. Empirical indices [i.e. Wet-Bulb Globe Temperature in $^{\circ }$C (WBGT)^26^ have been developed for specific and restricted thermal or meteorological conditions, such as indoor environments, or warm or cold environments. Empirical indices are regularly expressed through a simple formula and are not well suited for application to other thermal conditions or general use. Rational indices are calculated using complicated computational models based on a human energy equilibrium. A previous study^11^ proposed that certain rational thermal indices, such as Universal Thermal Climate Index in $^{\circ }$C (UTCI)^27,28^, Perceived Temperature in $^{\circ }$C (PT)^29^, Physiologically Equivalent Temperature in $^{\circ }$C (PET)^30^ and Outdoor Standard Effective Temperature in $^{\circ }$C (SET*)^31^, are effective for evaluating general thermal environments globally. These rational indices are too complex to be calculated with a simple equation by non-experts, e.g., urban planners and architects. Therefore, RayMan was developed as a Windows-based application to calculate mean radiant temperature in $^{\circ }$C ($T_{mrt}$) and these rational indices^32,33^. However, RayMan is compiled in Delphi, and is thus only supported on Windows 7 or older versions, and thus is not under active maintenance or development. Therefore, RayMan has failed to keep pace with advances in computational technology and thermo-physiological knowledge, and thus cannot be applied to innovative thermal indices, such as an improved version of the modified Physiologically Equivalent Temperature in $^{\circ }$C (mPET)^34–36^. This raises the need for a successor application for innovative thermal indices or further modification of existing thermal indices. A former Python package (pythermalcomfort) was proposed to calculate thermal comfort indices including emblematically empirical and rational indices using essential simulation of the $T_{mrt}$ based on the RayMan routine as inputs^37^. The other two Python packages—Ladybug and thermofeel^38^—for calculating thermal indices mainly and originally focused on UTCI. Thermofeel aims to apply outputs of the traditional weather numeric model to calculate $T_{mrt}$ as the input for calculating UTCI, while Ladybug-tool applies 3-dimensional street canyon and building to simulate $T_{mrt}$. However, the results of Ladybug tool are different from ENVI-met^39,40^ and field measurements^41^. These three Python packages are all unable to calculate mPET. Thus, a novel Python library is proposed to calculate rational thermal indices—specifically advanced version of the mPET, directly simulate $T_{mrt}$, and serve as an inheritor of RayMan.

## Human biometeorological library in Python

The Python library is named “biometeo” as an abbreviation of “human biometeorology”. This section first describes the biometeo library functions, followed by an explanation of the library installation and its input and output variables.

### Biometeo library functions

Table 1 summarizes the biometeo library functions, categorized as the auxiliary functions and computational models of human biometeorological indices. The biometeo library mainly provides the computational models of human biometeorology to calculate PET^30^, Predicted Mean Vote (PMV)^42^, UTCI^27,28^ based on Fiala-model^43–45^, SET*^31^ and specifically an innovative thermal index—mPET^34–36^. Beside of air temperature in $^{\circ }$C ($T_{a}$), calculating these thermal indices requires $T_{mrt}$, vapor pressure in hPa (VP), and wind speed at the height of 1.1 m in m/s ($v_{1.1\textrm{m}}$) along with basic input variables in the biometeo Python package. These necessary input variables are not always available by regular measurements. The original UTCI requires wind speed at the height of 10 m in m/s ($v_{10\textrm{m}}$) as one of standard input variable, which is different from the other thermal indices. The input of wind speed variable of UTCI in biometeo package is $v_{1.1\textrm{m}}$. This will be automatically adjusted with a wind speed exponential function to $v_{10\textrm{m}}$ in the biometeo package^46^. This unified the input variables for users to calculate thermal indices. $T_{mrt}$ can be calculated by two indirect measurement approaches using black globe temperature^47^ or six-directional radiation fluxes^48^. However, these two measurements are not regular meteorological measurement variables. Thus, $T_{mrt}$ is simulated by several different approaches. The simplest approach is only the given solar constant which is related to the local target time and coordinate information including longitude, latitude, and elevation above sea level. The more accurate approach adds global radiation or cloud cover ratio as a variable, while the third approach also includes respectively or assembly additional variables, such as sky view factor, diffuse radiation, and fish eye photo. The Windows-based RayMan application was designed to simulate $T_{mrt}$^32, 33^. These three approaches to simulate $T_{mrt}$ are provided by RayMan and now inherited by biometeo except fisheye photo approach. Otherwise, $v_{1.1\textrm{m}}$is needed to calculate thermal indices. Regular wind speed measurements are taken at a height of 10 m, based on recommendations from the World Meteorological Organization^49^. This requires an additional function to deduce wind speed from 10 to 1.1 m^46^. Thermal indices are commonly calculated using vapor pressure, relative humidity or both. Most datasets or reporting stations only provide vapor pressure or relative humidity, but both may be required to calculate the thermal index. Finally, these three additional functions are appended in the biometeo library.

**Table 1:** Biometeo library functions.

Auxiliary functions for human biometeorological variables	
$T_{mrt}$_calc	Calculation of $T_{mrt}$ respectively or assembly from coordinates and time of target location, solar radiation, cloud cover ratio or sky view factor
VP_RH_exchange	Exchange of VP and RH
$V_{1\textrm{m}}$_cal	Calculation of wind speed at the height of 1.1 m from any height

Computational models of thermal indices	
PMV	Calculation of predicted mean vote by human biometeorological variables
SET*	Calculation of outdoor standard effective temperature by human biometeorological variables
PET	Calculation of physiologically equivalent temperature by human biometeorological variables
mPET	Calculation of modified physiologically equivalent temperature by human biometeorological variables
UTCI	Calculation of universal thermal climate index by human biometeorological variables

### Biometeo library installation

The Python library biometeo is installed simply with a Python pip command as follow:

$$\begin{aligned} \boxed {{\text {pip3 install biometeo}}} \end{aligned}$$

Installation of the Python library biometeo requires Python versions 3.8, 3.9, or 3.10 running on Windows, Linux, or Mac OS. All installation information can also be found at:

$$\begin{aligned} \boxed {{\text {https://pypi.org/project/biometeo/}}} \end{aligned}$$

### Input and output variables of biometeo library

The input and output variables are numerous and complex in biometeo library. The explanation and abbreviation of the variables will be considered in the section.

#### Input and output variables for human biometeorological indices

The basic input variables for calculating rational thermal indices are the $T_{a}$, VP or relative humidity in % (RH), $v_{1.1\textrm{m}}$ or wind speed at the height of 10 m in m/s ($v_{10\textrm{m}}$), and $T_{mrt}$. The PMV, PET, and mPET require $T_{a}$, VP, $v_{1.1\textrm{m}}$, and $T_{mrt}$ as fundamental environmental inputs. SET* requires $T_{a}$, RH, $v_{1.1\textrm{m}}$, and $T_{mrt}$ as basic input. The original UTCI only applies $T_{a}$, VP, $v_{10\textrm{m}}$, and $T_{mrt}$ to assess the thermal perception. Hereby, UTCI in the biometeo package applies $v_{1.1\textrm{m}}$ . In addition to environmental variables, calculating thermal indices also requires thermo-physiological and textile variables. All of the above mentioned thermal indices except UTCI require identifying inputs for both the thermo-physiological and textile variables. The UTCI considers an idealized walking subject with corresponding clothing insulation of individual subject in clo ($I_{cl}$) to current $T_{a}$^50^. On contrary, SET* and PMV all require input of a certain $I_{cl}$. The $I_{cl}$ of the PET is fixed at 0.9 clo. The mPET can alternatively input individual $I_{cl}$ or apply $I_{cl}$ corresponding to the current $T_{a}$. The necessary thermo-physiological variables for SET* are activity loading of individual subject in W (work), height of individual subject in m (ht) and body weight of individual subject in kg (mbody). PMV also requires age of individual subject (age) and gender of individual subject (gender) as input. PET and mPET further require defining posture of the individual subject (pos). Briefly, $I_{cl}$, work, ht, mbody, age, gender, and pos are additional thermo-physiological and textile variables used as inputs in biometeo. The basic output of these thermal indices are effective temperature (SET* and UTCI), equivalent temperature (PET and mPET), or thermal perception level (PMV). PMV uses another two output variables (Teq) and heat loss through clothing (hclo)—to validate PMV as a reasonable result. The computational model of PET and mPET generate more thermo-physiological parameters and energy fluxes as outputs than the SET*-model. These thermo-physiological parameters and energy fluxes include core temperature of individual subject in $^{\circ }$C ($T_{core}$), skin temperature of individual subject in $^{\circ }$C ($T_{sk}$), wearing clothing temperature in $^{\circ }$C ($T_{cl}$), skin wettedness of subject (dimensionless) (wetsk), total metabolic rate in W (h), respiratory energy fluxes of subject in W ($E_{re}$), convective energy fluxes of subject in W ($c_{sum}$), radiative energy fluxes of subject in W ($r_{sum}$), skin diffuse energy fluxes of subject in W ($E_{d}$), and sweating evaporative energy fluxes of subject in W ($E_{sw}$) for PET. The additional thermo-physiology and energy fluxes output for mPET are listed as $T_{core}$, mean skin temperature of subject in $^{\circ }$C ($T_{sk, mm}$), mean wearing clothing temperature of subject in $^{\circ }$C ($T_{cl, mm}$), mean skin vapor pressure of subject in hPa ($VP_{ts, mm}$), mean skin wettedness of subject (dimensionless) ($sk_{wetted, mm}$), saturated ration of subject skin (dimensionless) ($wet_{sk}$), h, $E_{re}$, $c_{sum}$, $r_{sum}$, and all total evaporative energy fluxes of subject skin in W ($wet_{sum}$). Finally, to calculate PMV, UTCI, PET, and mPET the foundational inputs are four environmental variables. Other optional variables can be automatically obtained using default values or values provided by users. The following are practical examples of applying the biometeo computational model.

$$\begin{aligned} \boxed {\{PMV, Teq, hclo\} = biometeo.PMV(Ta=20.0, VP=12.5, v=0.1, Tmrt=20.0)} \end{aligned}$$

$$\begin{aligned} \boxed {SET^{*} = biometeo.SET(Ta=20.0, RH=50.0, v=0.1, Tmrt=20.0)} \end{aligned}$$

$$\begin{aligned} \boxed {UTCI = biometeo.UTCI(Ta=20.0, VP=12.5, v=0.5, Tmrt=20.0)} \end{aligned}$$

$$\begin{aligned} \boxed {\begin{aligned}&\{PET\_v, Tcore, Tsk, Tcl, wetsk, metabolic\_rate, respiratory\_flux, convective\_flux, \\&radiative\_flux, diffuse\_flux, sweating\_flux\}= biometeo.PET(Ta=20.0, VP=12.5, v=0.1, Tmrt=20.0, work=\\&80, gender=1) \end{aligned}} \end{aligned}$$

$$\begin{aligned} \boxed {\begin{aligned}&\{mPET, Tcore, Tsk\_mm, Tcl, vpts, wetsk, icl, sk\_wetted\_mm, metabolic\_rate,\\&wet\_sum, convective\_flux, radiative\_flux, respiratory\_flux, energy\_balance\} = biometeo.mPET(Ta=20.0, VP=\\&12.5, v=0.1, Tmrt= 0.5,, icl=1.8, clo\_auto=False) \end{aligned}} \end{aligned}$$

#### Input and output variables for auxiliary functions

VP_RH_exchange and $V_{1\textrm{m}}$_cal are two simple functions. VP_RH_exchange requires only $T_{a}$ and either VP or RH to calculate the other. $V_{1\textrm{m}}$_cal requires only wind speed and the height of wind measurement to generate the $v_{1.1\textrm{m}}$. The two functions can be applied as in the following examples.

$$\begin{aligned} \boxed {\{VP\} = biometeo.VP\_RH\_exchange(Ta=20.0, RH=50.0)} \end{aligned}$$

or

$$\begin{aligned} \boxed {\{RH\} = biometeo.VP\_RH\_exchange(Ta=20.0, VP=12.5)} \end{aligned}$$

and

$$\begin{aligned} \boxed {v_{1.1\textrm{m}} = biometeo.v1\textrm{m}\_cal(WS=8.5, height=60)} \end{aligned}$$

The more complex of the three auxiliary functions is $T_{mrt}$_calc. The input variables of $T_{mrt}$_calc function include local time in floating hour format (hour_of_day), time zone offset (timezone_offset), longitude in ± 180 $^{\circ }$ (longitude), latitude in ± 90 $^{\circ }$ (latitude), sea level height in m (sea_level_height), day of the year (day_of_year), $T_{a}$, VP, RH, $v_{1.1\textrm{m}}$, cloud cover in 0–8 octals (N), global radiation in W/m$^{2}$ (G), ratio of diffuse and global radiation (dimensionless) (DGratio), surface temperature in $^{\circ }$C ($T_{ob}$), Linke turbidity (dimensionless) (ltf), sky view factor in 0.0–1.0 (OmegaF), albedo of the surrounding (dimensionless) (alb), albedo of the human being (dimensionless) (albhum), reduction of G presetting by obstacles in boolean (RedGChk), lower limit of RH for full diffuse radiation in % (foglimit), and Bowen ratio (dimensionless) (bowen). The output of the $T_{mrt}$_calc function includes maximum of directional solar radiation in W/m$^{2}$ (Imax), maximum of global radiation in W/m$^{2}$ (Gmax), maximum of diffuse solar radiation in W/m$^{2}$ (Dmax), current directional solar radiation in W/m$^{2}$ (Itat), current global radiation in W/m$^{2}$ (Gtat), current diffuse solar radiation in W/m$^{2}$ (Dtat), atmospheric long wave radiation in W/m$^{2}$ (A), long wave radiation from surface in W/m$^{2}$ (Eu), long wave radiation from sides in W/m$^{2}$ (Es), and the most important variable—$T_{mrt}$. A following simple case calculates the current $T_{mrt}$ for a given $T_{a}$, RH, $v_{1.1\textrm{m}}$, and longitude, latitude, and the sea level height. The other necessary inputs are automatically set up as default values.

$$\begin{aligned} \boxed {\begin{aligned}&\{Tmrt, VP, Imax, Gmax, Dmax, Itat, Gtat, A, Eu, Es, Tob\} = biometeo.Tmrt\_calc(Ta=20.0, RH=50.0, v=\\&0.1, longitude=121.5, latitude=23.5, sea\_level\_height=30) \end{aligned}} \end{aligned}$$

Calculation of $T_{mrt}$ at specified time requires hour_of_day, day_of_year, and timezone_offset. Otherwise, G, N, DGratio, and OmegaF are commonly used optional inputs to calculate precise $T_{mrt}$ under specifically spatial and temporal conditions. Hereby, OmegaF should be applied with shadowed or non-shadowed conditions. Therefore, OmegaF can be only fixed in 1.0 in $T_{mrt}$_calc. See the following example:

$$\begin{aligned} \boxed {\begin{aligned}&\{Tmrt, VP, Imax, Gmax, Dmax, Itat, Gtat, A, Eu, Es, Tob\} = biometeo.Tmrt\_calc(Ta=20.0, RH=50.0, v=\\&0.1, longitude=121.5, latitude=23.5, sea\_level\_height=30, hour\_of\_day=15.8, day\_of\_year=\\&210, timezone\_offset=8, N=6) \end{aligned}} \end{aligned}$$

All of the necessary inputs, optional inputs, default, and outputs of the functions and thermal indices are summarized in Table 2.

**Table 2:** Inputs and outputs of biometeo package.

Auxiliary functions	Fundmental inputs	Optional inputs	Defaults	Outputs
$T_{mrt}$_calc	Ta, RH, v ($v_{1.1\textrm{m}}$), longitude, latitude, sea_level_height	day_of_year, hour_of_day, timezone_offset, N, G, DGratio, Tob, ltf, alb, albhum, RedGChk, foglimit, bowen	Now time, N $=$ 0, OmegaF $=$ 1.0, alb $=$ 0.3, albhum $=$ 0.3, RedGChk $=$ False, foglimit $=$ 90, bowen $=$ 1.0	{Tmrt, VP, Imax, Gmax, Dmax, Itat, Gtat, A, Eu, Es, Tob}
VP_RH_exchange	Ta, VP or RH			{VP} or {RH}
$V_{1\textrm{m}}$_cal	WS, height			v ($v_{1.1\textrm{m}}$)
Thermal indices				
PMV	Ta, VP, v ($v_{1.1\textrm{m}}$), Tmrt	icl, work, ht, mbody, age, sex	icl $=$ 0.6, work $=$ 80, ht $=$ 1.75, mbody $=$ 75, age $=$ 35, sex $=$ 1 (male)	{PMV, Teq, hclo}
SET*	Ta, RH, v ($v_{1.1\textrm{m}}$), Tmrt	icl, work, ht, mbody	icl $=$ 0.9, work $=$ 80, ht $=$ 1.75, mbody $=$ 75	SET*
PET	Ta, VP, v ($v_{1.1\textrm{m}}$), Tmrt	icl, work, ht, mbody, age, sex, pos	icl $=$ 0.9, work $=$ 80, ht $=$ 1.75, mbody$=$75, age $=$ 35, sex $=$ 1 (male), pos $=$ 1 (stand)	{PET, Tcore, Tsk, Tcl, wetsk, metabolic_rate, respiratory_flux, convective_flux, radiative_flux, diffuse_flux, sweating_flux}
mPET	Ta, VP, v ($v_{1.1\textrm{m}}$), Tmrt	icl, work, ht, mbody, age, sex, pos, auto_clo	icl $=$ 0.9, work $=$ 80, ht $=$ 1.75, mbody $=$ 75, age $=$ 35, sex $=$ 1 (male), pos $=$ 1 (stand), auto_clo $=$ True	{mPET, Tcore, Tsk_mm, Tcl, vpts, wetsk, icl, sk_wetted_mm, metabolic_rate, wet_sum, convective_flux, radiative_flux, respiratory_flux, energy_balance}
UTCI	Ta, VP, v ($v_{1.1\textrm{m}}$), Tmrt			UTCI

## Comparison of biometeo and RayMan

**Figure 1:**
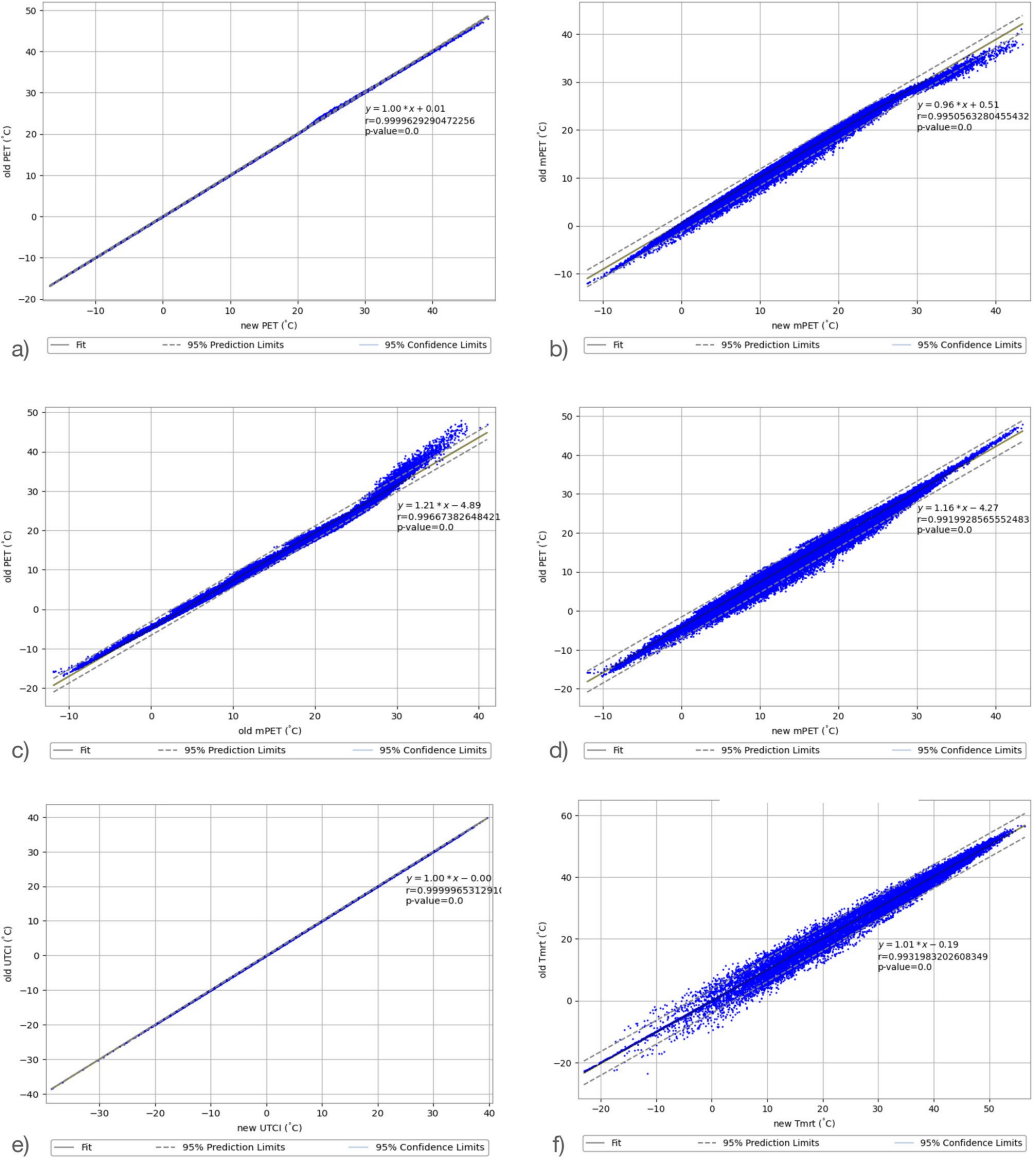
Applying regularly measured meteorological data from Freiburg weather stations from 1999 to 2004 to compare (**a**) PET from RayMan (old) and biometeo (new), (**b**) mPET from RayMan (old) and biometeo (new), (**c**) PET and mPET from RayMan (old), (**d**) PET from RayMan (old) and mPET from biometeo (new), (**e**) UTCI from RayMan (old) and biometeo (new), and (**f**) $T_{mrt}$ from RayMan (old) and biometeo (new).

Comparisons between RayMan and biometeo in PET, mPET, UTCI, and $T_{mrt}$, applying old data set generated by original RayMan in Freiburg from 1999 to 2004, are shown in Fig. 1. It is hardly feasible to get the hardware and old-version Windows to recalculate PMV and SET* by RayMan. Also, we have no previous datasets to be able to validate them. Figure 1a and e show that biometeo is not significantly different from RayMan to calculate PET and UTCI. On contrary, the mPET which results from biometeo is slightly different from the mPET from RayMan. The obvious difference occurs, while the mPET from RayMan is higher than about 28 $^{\circ }$C (Fig. 1b). Comparing the mPETs from RayMan and biometeo to the PET from RayMan, we can find that the distribution of xy-paring in mPET from biometeo versus PET from RayMan are different from other one (Fig. 1c,d). The reasonable causes are that Delphi has no matrices operation libraries and sufficiently accurate floating point arithmetic, while Python has Numpy library and advanced precision in floating-point operations. The difference between T_mrt_ from RayMan and that from biometeo may also be caused by the similar reasons (Fig. 1f). Calculating $T_{mrt}$ requires solar zenith and trigonometric functions. Python has better trigonometric functions supported by Numpy than Delphi. The difference of $T_{mrt}$ occurs regularly at the moments of sunrise and sunset. This could be caused by the operations of trigonometric functions and if-loop. Overall, biometeo can generate almost the same PET and UTCI as RayMan. The mPET from biometeo was further revised as an more appropriate thermal index than from RayMan. The $T_{mrt}$ generated by biometeo is applicable. However, the precision of $T_{mrt}$ from biometeo should further validated to the observation data.

## Example of use

**Figure 2:**
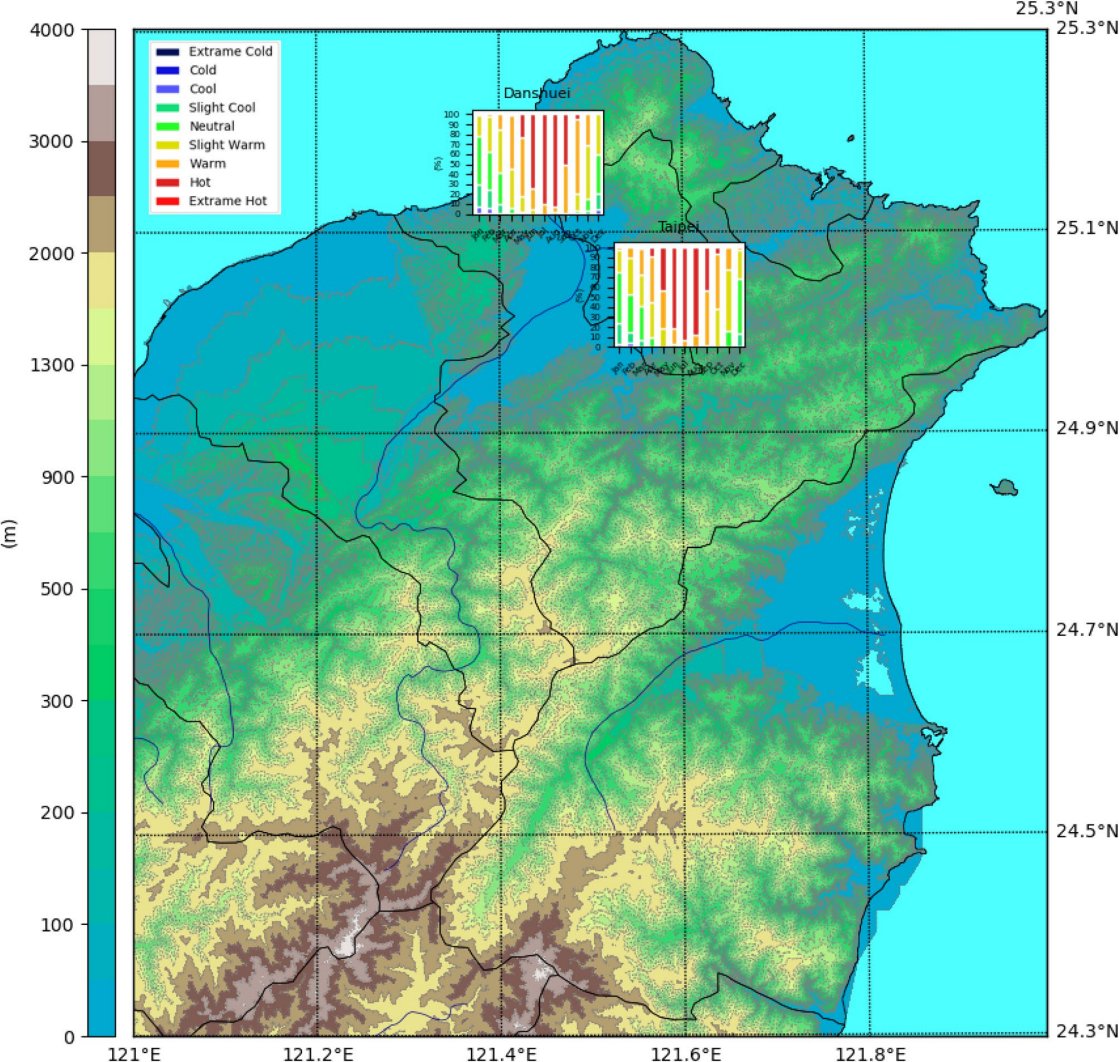
A case study applying regularly measured meteorological data from Taipei and Danshuei weather stations from 2011 to 2016 to investigate the probability of monthly thermal conditions.

**Figure 3:**
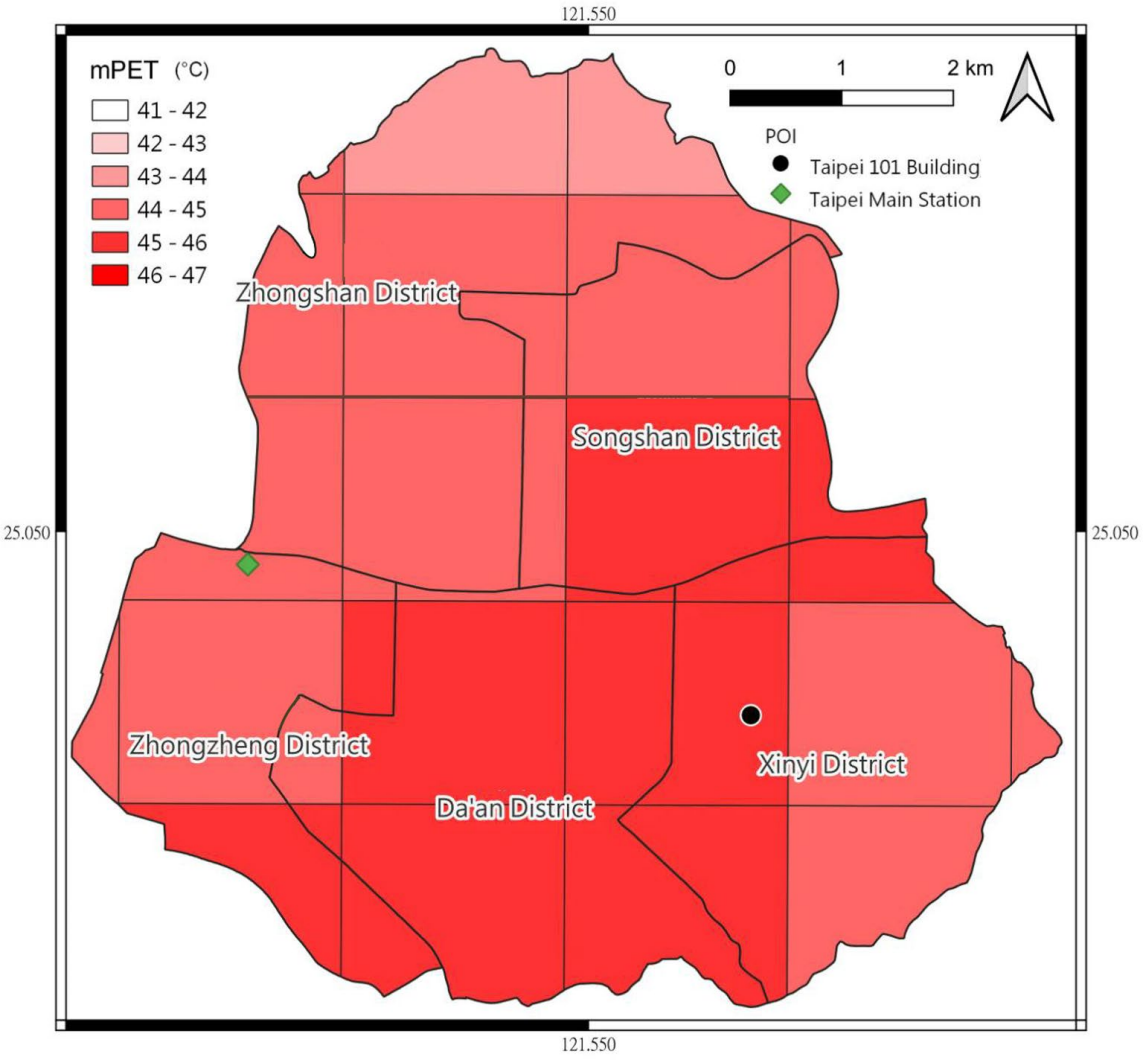
Reanalyzing monthly mean data for July 2011 using weather regional and forecast model downscaling to consider geographically thermal condition patterns, shown in Mercator projection overlaid with urban administration districts.

This section provides two examples to illustrate the application of biometeo integrated with the other Python packages for scientific visualization. The first example uses regularly measured meteorological data from Taiwan’s Central Weather Bureau (CWB) to investigate the thermal environment of north Taiwan. The CWB data follows the measurement rules of the World Meteorological Organization^49^. We apply the $T_{a}$, $v_{10\textrm{m}}$, RH, N, and also the geographic information of weather stations to calculate the mPET with VP_RH_exchange, $V_{1\textrm{m}}$_cal, $T_{mrt}$_calc, and mPET Python module in the biometeo library, with Fig. 2. This is the monthly probability of thermal perception in two stations—Taipei and Danshuei—in north Taiwan during 2011 to 2016 on a topography map. The north Taiwan is classified as Cfa classification according to the Köppen–Geiger climate map^51,52^. The Fig. 2 showning rsults for the monthly probability of thermal perception in two north Taiwan stations—Taipei and Danshuei from 2011 to 2016. The figure is visualized using the matplotlib Python package implemented using basemap and gdal Python packages. This kind of visualization can be used to simply and effectively investigate seasonal differences of thermal stress in different regions. Figure 3 shows the thermal sensation in a grid format on an administration distinct map. The mPET on this map is calculated using biometeo with meteorological data from the Weather Research and Forecasting (WRF) Model^53–55^. A previous study applied RayMan as an external support on a Windows routine to calculate $T_{mrt}$ and mPET with WRF output on a Linux sever^56^. In this case, global radiation from WRF was withdrawn by netCDF4 Python package and directly applied as Gtat to be inserted into the $T_{mrt}$_calc of the biometeo library to calculate the $T_{mrt}$. Afterward, mPET was generated by inserting $T_{a}$, VP, $v_{1.1\textrm{m}}$, and $T_{mrt}$ into the mPET computational model. Finally, a visualization thermal perception map was generated (Fig. 3). These two cases show the potential for the interdisciplinary application of biometeo to investigate human biometeorology.

## Discussion and summary

RayMan was previously an effective, widely applicable, and convenient method for calculating rational thermal indices, including most of the most widely used indices, such as PET, PMV, PT, SET* and UTCI^7,11,57^. RayMan could simulate $T_{mrt}$ through several approaches, such as only given coordinates and target time, or any additional term from G, DGratio, N, OmegaF, and fisheye photo or building obstacle map^32,33^. Although previous studies^58,59^ critiqued the precision of $T_{mrt}$ simulations by RayMan, convenience and various modeling conditions made RayMan a multifunctional and multipurpose software for investigating human biometeorology, thermal perception, urban climates, and tourism. However, due to update and maintenance issues related to Delphi, RayMan updates and support terminated after Windows 7. The proposed biometeo library recapitulates parts of RayMan functionality in Python to calculate thermal indices and simulate $T_{mrt}$. The major contributions of biometeo are the computational model and calculation processes for the innovative thermal index—mPET^34–36^. mPET is currently the only practical thermal index based on the dynamic thermo-physiological model and semi-steady state for the evaluation of thermal perception. The mPET model in biometeo is improved for effective computation and a comprehensive and realistic thermal index. To simulate $T_{mrt}$, biometeo avoids complicated approaches involving the application of fisheye photos or building obstacle maps. However, the $T_{mrt}$ generated by biometeo is slightly different by RayMan in sunset and sunrise moment. This should be further validated. Another Python package—pythermalcomfort—underperforms in terms of simulating $T_{mrt}$and requires additional external function support to obtain $T_{mrt}$, despite containing numeric models of PMV, Standard Effective Temperature in $^{\circ }$C (SET), UTCI, PET, WBGT, and other empirically thermal indices^37^. Thermofeel focuses on connection to the outputs of weather numeric model to calculate $T_{mrt}$ and UTCI^38^. Ladybug-tool has been shown with lots of uncertainties to evaluate thermal perception^41^. Future work will append new functions and input methods to simulate $T_{mrt}$ in biometeo, using improved image processing from fisheye photo and integrating Geographic Information System (GIS) data through GIS libraries in Python. Moreover, Python-based biometeo can be used to bridge various input file formats and datasets, such as simple text or csv files (pandas), complex netCDF-formats (netCDF4), and SQL-database (pyodbc), allowing for improved computational efficiency across different operating systems to generate useful scientific images and visualizations. Finally, future developments in advanced thermo-physiological mechanisms will allow for continuous improvement and development of mPET and other thermal indices. In summary, the Python package biometeo is a widely applicable, convenient and comprehensive tool to calculate effectively rational thermal indices for assessing thermal perception for application in urban climate and sustainable urban design, tourism, and public health across various spatial and temporal scales. Most importantly, biometeo is the only Python package with sufficient supporting tools for calculating mPET.

### Supplementary Information

Supplementary Information 1.

Supplementary Information 2.

## Data Availability

All data generated or analysed during this study are included in this published article and its Supplementary information files.

## Abbreviations

A: Atmospheric long wave radiation in W/m$^{2}$
age: Age of individual subject
alb: Albedo of the surrounding (dimensionless)
albhum: Albedo of the human being (dimensionless)
bowen: Bowen ratio (dimensionless)
$c_{sum}$: Convective energy fluxes of subject in W
DGratio: Ratio of diffuse and global radiation (dimensionless)
Dmax: Maximum of diffuse solar radiation in W/m$^{2}$
Dtat: Current diffuse solar radiation in W/m$^{2}$
$E_{d}$: Skin diffuse energy fluxes of subject in W
$E_{re}$: Respiratory energy fluxes of subject in W
Es: Long wave radiation from sides in W/m$^{2}$
$E_{sw}$: Sweating evaporative energy fluxes of subject in W
Eu: Long wave radiation from surface in W/m$^{2}$
foglimit: Lower limit of RH for full diffuse radiation in %
G: Global radiation in W/m$^{2}$
gender: Gender of individual subject
Gmax: Maximum of global radiation in W/m$^{2}$
Gtat: Current global radiation in W/m$^{2}$
h: Total metabolic rate in W
ht: Height of individual subject in m
$I_{cl}$: Clothing insulation of individual subject in clo
Imax: Maximum of directional solar radiation in W/m$^{2}$
Itat: Current directional solar radiation in W/m$^{2}$
ltf: Linke turbidity (dimensionless)
mbody: Body weight of individual subject in kg
mPET: modified Physiologically Equivalent Temperature in $^{\circ }$C
N: Cloud cover in 0–8 octals
OmegaF: Sky view factor in 0.0–1.0
PET: Physiologically Equivalent Temperature in $^{\circ }$C
PT: Perceived Temperature in $^{\circ }$C
PMV: Predicted Mean Vote
pos: Posture of the individual subject
$r_{cl}$: Vapor resistance of clothing only for sweating evaporation in hPa m$^{2}$/W
RH: Relative humidity in %
$r_{sum}$: Radiative energy fluxes of subject in W
SET: Standard Effective Temperature in $^{\circ }$C
SET*: Outdoor Standard Effective Temperature in $^{\circ }$C
$sk_{wetted, mm}$: Mean skin wettedness of subject (dimensionless)
$T_{a}$: Air temperature in $^{\circ }$C
$T_{cl}$: Wearing clothing temperature in $^{\circ }$C
$T_{cl, mm}$: Mean wearing clothing temperature of subject in $^{\circ }$C
$T_{core}$: Core temperature of individual subject in $^{\circ }$C
$T_{mrt}$: Mean radiant temperature in $^{\circ }$C
$T_{ob}$: Surface temperature in $^{\circ }$C
$T_{sk}$: Skin temperature of individual subject in $^{\circ }$C
$T_{sk, mm}$: Mean skin temperature of subject in $^{\circ }$C
UTCI: Universal Thermal Climate Index in $^{\circ }$C
$v_{1.1\textrm{m}}$: Wind speed at the height of 1.1 m in m/s
$v_{10m}$: Wind speed at the height of 10 m in m/s
VP: Vapor pressure in hPa
$VP_{ts, mm}$: Mean skin vapor pressure of subject in hPa
WBGT: Wet-Bulb Globe Temperature in $^{\circ }$C
wetsk: Skin wettedness of subject (dimensionless)
$wet_{sk}$: Saturated ration of subject skin (dimensionless)
$wet_{sum}$: Total evaporative energy fluxes of subject skin in W
work: Activity loading of individual subject in W
$w_{sum}$: Water volume loss from subject in W

## References

[1] Mills G (2004). Urban Climate, Weather and Sustainability.

[2] Holmér I (2010). Climate change and occupational heat stress: Methods for assessment. Glob. Health Act..

[3] Höppe P (1997). Aspects of human biometeorology in past, present and future. Int. J. Biometeorol..

[4] Matzarakis A, Cheval S, Lin TP, Potchter O (2021). Challenges in applied human biometeorology. Atmosphere.

[5] Parsons K (2010). Thermal Comfort in Buildings.

[6] Parsons K (2013). Occupational health impacts of climate change: Current and future ISO standards for the assessment of heat stress. Ind. Health.

[7] Vanos JK, Warland JS, Gillespie TJ, Kenny NA (2010). Review of the physiology of human thermal comfort while exercising in urban landscapes and implications for bioclimatic design. Int. J. Biometeorol..

[8] VDI. VDI 3787 environmental meteorology methods for the human biometeorological evaluation of climate and air quality for urban and regional planning at regional level part I: Climate (2008).

[9] Yildiz ND, Avdan U, Yilmaz S, Matzarakis A (2018). Thermal map assessment under climate and land use changes; a case study for Uzundere basin. Environ. Sci. Pollut. Res..

[10] Parsons K (2002). Human Thermal Environments.

[11] Staiger H, Laschewski G, Matzarakis A (2019). Selection of appropriate thermal indices for applications in human biometeorological studies. Atmosphere.

[12] Nikolopoulou M, Lykoudis S (2007). Use of outdoor spaces and microclimate in a Mediterranean urban area. Build. Environ..

[13] Lenzholzer S (2010). Engrained experience-a comparison of microclimate perception schemata and microclimate measurements in Dutch urban squares. Int. J. Biometeorol..

[14] Lenzholzer S, van der Wulp NY (2010). Thermal experience and perception of the built environment in Dutch urban squares. J. Urban Des..

[15] Vasilikou C, Nikolopoulou M (2020). Outdoor thermal comfort for pedestrians in movement: Thermal walks in complex urban morphology. Int. J. Biometeorol..

[16] Krüger E (2017). Impact of site-specific morphology on outdoor thermal perception: A case-study in a subtropical location. Urban Clim..

[17] Manavvi S, Rajasekar E (2020). Semantics of outdoor thermal comfort in religious squares of composite climate: New Delhi, India. Int. J. Biometeorol..

[18] Klemm W, Heusinkveld BG, Lenzholzer S, Jacobs MH, Hove BV (2015). Psychological and physical impact of urban green spaces on outdoor thermal comfort during summertime in the Netherlands. Buildi. Environ..

[19] Manavvi S, Rajasekar E (2021). Evaluating outdoor thermal comfort in “haats”—The open air markets in a humid subtropical region. Build. Environ..

[20] Manavvi S, Rajasekar E (2022). Evaluating outdoor thermal comfort in urban open spaces in a humid subtropical climate: Chandigarh, India. Build. Environ..

[21] Nouri AS, Lopes A, Costa JP, Matzarakis A (2018). Confronting potential future augmentations of the physiologically equivalent temperature through public space design: The case of Rossio, Lisbon. Sustain. Cities Soc..

[22] Oke TR, Mills G, Christen A, Voogt JA (2017). Urban Climates.

[23] ASHRAE. Ansi/ashrae standard 55 thermal environmental conditions for human occupancy (2017).

[24] ISO. ISO 7730: Ergonomics of the thermal environment analytical determination and interpretation of thermal comfort using calculation of the PMV and PPD indices and local thermal comfort criteria. Management (2005).

[25] Epstein Y, Moran DS (2006). Thermal comfort and the heat stress indices. Ind. Health.

[26] Budd GM (2008). Wet-bulb globe temperature (WBGT)-its history and its limitations. J. Sci. Med. Sport.

[27] Bröde P (2012). Deriving the operational procedure for the universal thermal climate index (UTCI). Int. J. Biometeorol..

[28] Jendritzky G, de Dear R, Havenith G (2012). UTCI-why another thermal index?. Int. J. Biometeorol..

[29] Staiger H, Laschewski G, Grätz A (2012). The perceived temperature—a versatile index for the assessment of the human thermal environment. Part a: Scientific basics. Int. J. Biometeorol..

[30] Höppe P (1999). The physiological equivalent temperature—a universal index for the biometeorological assessment of the thermal environment. Int. J. Biometeorol..

[31] Gagge AP, Fobelets AP, Berglund LG (1986). Standard predictive index of human response to the thermal environment. ASHRAE Trans..

[32] Matzarakis A, Rutz F, Mayer H (2007). Modelling radiation fluxes in simple and complex environments—Application of the Rayman model. Int. J. Biometeorol..

[33] Matzarakis A, Rutz F, Mayer H (2010). Modelling radiation fluxes in simple and complex environments: basics of the Rayman model. Int. J. Biometeorol..

[34] Chen Y-C, Matzarakis A (2014). Modification of physiologically equivalent temperature. J. Heat Island Inst. Int..

[35] Chen Y-C, Matzarakis A (2018). Modified physiologically equivalent temperature-basics and applications for western European climate. Theor. Appl. Climatol..

[36] Chen Y-C, Chen W-N, Chou C, Matzarakis A (2020). Concepts and new implements for modified physiologically equivalent temperature. Atmosphere.

[37] Tartarini F, Schiavon S (2020). pythermalcomfort: A python package for thermal comfort research. SoftwareX.

[38] Brimicombe C (2022). Thermofeel: A python thermal comfort indices library. SoftwareX.

[39] Elwy I, Ibrahim Y, Fahmy M, Mahdy M (2018). Outdoor microclimatic validation for hybrid simulation workflow in hot arid climates against environment and field measurements. Energy Procedia.

[40] Bruse M, Fleer H (1998). Simulating surface–plant–air interactions inside urban environments with a three dimensional numerical model. Environ. Model. Softw..

[41] Evola G (2020). A novel comprehensive workflow for modelling outdoor thermal comfort and energy demand in urban canyons: Results and critical issues. Energy Build..

[42] Fanger PO (1972). Thermal Comfort: Analysis and Applications in Environmental Engineering.

[43] Fiala D, Lomas KJ, Stohrer M (1999). A computer model of human thermoregulation for a wide range of environmental conditions: The passive system. J. Appl. Physiol..

[44] Fiala D, Lomas KJ, Stohrer M (2001). Computer prediction of human thermoregulatory and temperature responses to a wide range of environmental conditions. Int. J. Biometeorol..

[45] Fiala D, Havenith G, Bröde P, Kampmann B, Jendritzky G (2012). UTCI-FIALA multi-node model of human heat transfer and temperature regulation. Int. J. Biometeorol..

[46] Matzarakis A, Rocco MD, Najjar G (2009). Thermal bioclimate in Strasbourg-The 2003 heat wave. Theor. Appl. Climatol..

[47] ISO. ISO 7726 ergonomics of the thermal environment—Instruments for measuring physical quantities. ISO Standard (1998).

[48] VDI. VDI 3787 part I: Environmental meteorology, methods for the human-biometeorological evaluation of climate and air quality for the urban and regional planning at regional level. Part I: Climate (1998).

[49] Jarraud M (2008). Guide to Meteorological Instruments and Methods of Observation (WMO-no. 8).

[50] Havenith G (2012). The UTCI-clothing model. Int. J. Biometeorol..

[51] Beck HE (2018). Present and future köppen–Geiger climate classification maps at 1-km resolution. Sci. Data.

[52] Kottek M, Grieser J, Beck C, Rudolf B, Rubel F (2006). World map of the köppen–Geiger climate classification updated. Meteorol. Z..

[53] Hersbach H (2020). The era5 global reanalysis. Q. J. R. Meteorol. Soc..

[54] Skamarock, W. C. *et al.* A description of the advanced research WRF version 3, NCAR technical note TN-475+STR (2008).

[55] Tsai I-Chun, Hsieh Pei-Rong, Cheng Chao-Tzuen, Tung Yu-Shiang, Lin Lee-Yaw, Hsu Huang-Hsiung (2022). Impacts of 2 and 4$$^{\circ }$$ C global warmings on extreme temperatures in Taiwan. Int. J. Climatol..

[56] Giannaros TM, Lagouvardos K, Kotroni V, Matzarakis A (2018). Operational forecasting of human-biometeorological conditions. Int. J. Biometeorol..

[57] Li, J. & Liu, N. The perception, optimization strategies and prospects of outdoor thermal comfort in china: A review (2020).

[58] Crank Peter J., Middel Ariane, Wagner Melissa, Hoots Dani, Smith Martin, Brazel Anthony (2020). Validation of seasonal mean radiant temperature simulations in hot arid urban climates. Sci. Total Environ..

[59] Thorsson S, Lindberg F, Eliasson I, Holmer B (2007). Different methods for estimating the mean radiant temperature in an outdoor urban setting. Int. J. Climatol..

